# Distinct types of multicellular aggregates in *Pseudomonas aeruginosa* liquid cultures

**DOI:** 10.1038/s41522-023-00412-5

**Published:** 2023-07-28

**Authors:** Gavin Melaugh, Vincent A. Martinez, Perrin Baker, Preston J. Hill, P. Lynne Howell, Daniel J. Wozniak, Rosalind J. Allen

**Affiliations:** 1 SUPA, School of Physics and Astronomy, University of Edinburgh, Edinburgh, EH9 3FD UK; 2 School of Engineering, University of Edinburgh, Edinburgh, EH9 3JL UK; 3 Program in Molecular Medicine, The Hospital for Sick Children, Toronto, M5G 0A4 ON Canada; 4 Departments of Microbial Infection and Immunity, Microbiology, Infectious Diseases Institute, Ohio State University, Columbus, OH 43210 USA; 5 Department of Biochemistry, University of Toronto, Toronto, M5S 1A8 ON Canada; 6 Theoretical Microbial Ecology, Institute of Microbiology, Faculty of Biological Sciences, Friedrich Schiller University, Jena, 07745 Germany

**Keywords:** Biofilms, Applied microbiology

## Abstract

*Pseudomonas aeruginosa* forms suspended multicellular aggregates when cultured in liquid media. These aggregates may be important in disease, and/or as a pathway to biofilm formation. The polysaccharide Psl and extracellular DNA (eDNA) have both been implicated in aggregation, but previous results depend strongly on the experimental conditions. Here we develop a quantitative microscopy-based method for assessing changes in the size distribution of suspended aggregates over time in growing cultures. For exponentially growing cultures of *P. aeruginosa* PAO1, we find that aggregation is mediated by cell-associated Psl, rather than by either eDNA or secreted Psl. These aggregates arise de novo within the culture via a growth process that involves both collisions and clonal growth, and Psl non-producing cells do not aggregate with producers. In contrast, we find that stationary phase (overnight) cultures contain a different type of multicellular aggregate, in which both eDNA and Psl mediate cohesion. Our findings suggest that the physical and biological properties of multicellular aggregates may be very different in early-stage vs late-stage bacterial cultures.

## Introduction

Bacteria tend to aggregate when suspended in liquid or viscoelastic media. In clinical settings, this can have devastating consequences. For example, in the cystic fibrosis lung, aggregates of the opportunistic pathogen *Pseudomonas aeruginosa* are implicated in life-long lung infections that can result in complete failure of the respiratory system^1–3^. Bacterial aggregation is also relevant in wastewater treatment where pollutant-degrading bacteria form compact aggregates (or flocs)^4^; here, unpredictability in floc formation is economically costly^5^. From a microbiological perspective, aggregates can show similar properties to surface-attached biofilms, including antibiotic tolerance^6–8^, and in some cases they may initiate biofilm formation^9,10^. Yet despite its importance, a clear picture of the mechanisms underlying aggregate formation under different environmental conditions remains lacking.

In this work, we investigate quantitatively the aggregation of the *P. aeruginosa* lab strain PAO1 in liquid culture. We compare aggregates formed during exponential growth with those in overnight, stationary phase cultures. *P. aeruginosa* is widely used as a model organism for biofilm formation on surfaces^11–15^, but it also forms aggregates in liquid culture^6,7,9,16,17^. These aggregates have been observed, in different studies, in both exponential and stationary phase cultures^6,7,9,16,17^, with most reports focusing on late-log or stationary phase^6,9,16,17^. It is unclear whether differences exist between aggregates that form in different growth phases. In late stationary phase, aggregate dispersal has been reported^17^ (in a similar manner to biofilm dispersal^18^).

Extracellular polymeric substances (EPS) play a central role in the multicellular behaviour of *P. aeruginosa*^19–24^. For the PAO1 strain, three polymers have been shown to be important in the formation of biofilms on surfaces: the two polysaccharides Psl^25–29^ and Pel^27,28^, and extracellular DNA (eDNA)^16,26,27,30,31^ (in other strains, alginate production is also a significant factor). The protein CdrA has also been implicated in biofilm formation through its propensity to bind to Psl and Pel^32,33^. Of the three polymers, Psl is thought to be the predominant biofilm matrix component for the PAO1 strain^34^, facilitating cell-surface and cell–cell adhesion^29,35^, as well as providing mechanical stability and structural integrity to the biofilm^26^. Psl is an uncharged polymer of mannose, rhamnose, and glucose^26,36^ that exists in two forms^36–38^: a cell-free form that is secreted into the medium, and a cell-associated form that remains bound to the cell surface. It is not yet known which of these two forms predominates in the biofilm matrix. *P. aeruginosa* also produces a glycoside hydrolase, PslG, that is thought to play a regulatory role in Psl production^38^. PslG has been shown to specifically degrade Psl and to disrupt *P. aeruginosa* biofilms^39,40^. The Pel polysaccharide, composed of galactosamine and glucosamine sugar residues, is cationic^37^. Pel plays a more major role in biofilm formation for the PA14 strain (which is deficient in Psl production due to mutations in the *psl* genes), where it is required for biofilm maturation; however in PAO1 strains that are deficient in Psl production, upregulation of Pel can restore biofilm formation^41^. eDNA has been found to play a structural role in young PAO1 biofilms and is also present in the matrix of established biofilms^31^, although it seems to be less important for cell–cell cohesion than Psl^30^. eDNA is generated in biofilms, and in late-log phase liquid cultures, via lysis of a subpopulation of bacteria, in a manner that depends on quorum-sensing, flagella and pili^16,42^.

Perhaps unsurprisingly, EPS has also been implicated in the liquid-phase aggregation of *P. aeruginosa* cells^6,7,16,17^. In late-stage (late-log or stationary phase) cultures, aggregation has been found to be mediated by eDNA^16,17^, released in a quorum-sensing mediated manner^16^, or by a combination of eDNA and Psl^6,7^. It is not clear whether the aggregates are primarily formed by collisions between individual planktonic cells, or via clonal growth of a few aggregated cells, although for exponential-phase aggregates there is some evidence for a collision model^7^. So far only Kragh et al. have reported on aggregation in exponential phase cultures^7^, where Psl-mediated aggregates were observed. A strong dependence on inoculation conditions suggested that these exponential phase aggregates might be seeded by pre-existing aggregates in the inoculum^7^. Taken together, previous work suggests that exponential phase aggregates may be distinct from those found in late-log or stationary phase, but the details of how aggregates initiate and grow remain unclear.

The involvement of EPS in the multicellular behaviour of *P. aeruginosa* raises interesting socio-evolutionary questions^43–46^, since secreted EPS is shared between cells and could in principle provide a benefit to non-producing “cheaters”. Previous work on surface-attached biofilms has suggested that Psl production is a “social but non-cheatable” biofilm trait^43^. In other words, the benefits of Psl are shared between cells, but non-producers do not exploit these benefits. Investigating the socio-evolutionary implications of EPS in liquid-phase aggregates is of great interest, and may have wider relevance since aggregate formation has been suggested as a first step towards evolution of multicellular behaviour more generally^47,48^.

Aggregation in bacterial suspensions can be quantified via several methods. Probably the simplest method involves comparing the optical density of an unaggregated sample to that of the supernatant of an aggregated sample in which the aggregates have sedimented^49–51^. This gives information on the extent of aggregation, but not on the sizes or shapes of the aggregates. Advanced automated methods such as laser-diffraction analysis^17,52^, Coulter counting (electrical impedance method)^53^ and flow cytometry^54–56^ can be used to quantify aggregate sizes but these may require extra treatment steps before measurement, and do not measure aggregate shape. Microscopic imaging provides a direct readout of aggregate size and shape, and is often used to complement other methods. Here, as in previous studies^7,57^, we utilise microscopic imaging in a simple and quantitative manner to directly compute aggregate size distributions.

We observe distinct types of aggregates in exponential-phase cultures compared to overnight stationary-phase cultures. We show that cell-associated Psl mediates aggregation in exponential-phase cultures, while eDNA plays no significant role. We find evidence that aggregates appear de novo in these cultures, and grow via a mixture of collisions and clonal growth. In mixed cultures of Psl producers and non-producers, we find that Psl non-producers do not aggregate with producers, suggesting that Psl may be “non-cheatable”. In contrast, we find a different type of aggregate in overnight stationary phase cultures. Both Psl and eDNA are involved in these stationary-phase aggregates, but eDNA alone is sufficient to mediate aggregation. Our work helps clarify the nature of aggregation in early-stage exponential cultures and highlights the fact that different aggregation mechanisms are relevant under different conditions of bacterial growth.

## Results

### PAO1 forms aggregates during exponential growth

**Fig. 1:**
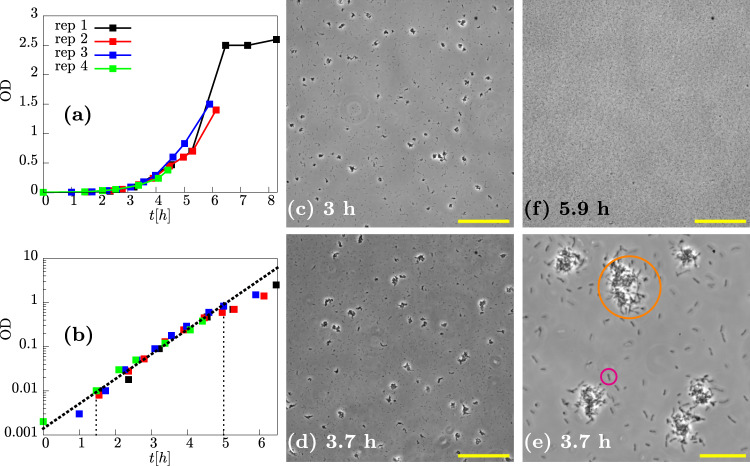
Aggregated and non-aggregated cells of PAO1 are present in the suspension during exponential growth. **a** Optical density (OD) as a function of time in four replicate (rep) experiments. **b** Data from (**a**) plotted on a logarithmic scale (*Y* axis), and line of best fit (dashed black line) corresponding to a growth rate of ~1.3 h^−1^ (doubling time of ~30 min). **c**–**f** Representative phase-contrast microscopy images at different times during growth. **c** 3 h, ×20 magnification, scale bar = 150 μm. **d** 3.7 h, ×20 magnification, scale bar = 150 μm. **e** 3.7 h, ×90 magnification, scale bar = 30 μm. Note the image in (**e**) was sampled from a different field of view to that in (**d**). **f** 5.9 h, ×20 magnification, scale bar = 150 μm. Orange and pink circles in (**e**) highlight an aggregate and single cell, respectively.

To assess the formation of *P. aeruginosa* aggregates during exponential growth, we inoculated liquid LB media with a low density of cells taken from an overnight culture, and took regular samples for microscopy while tracking the growth of the cultures via optical density (OD) measurements (Fig. 1a; see “Methods”). We observed exponential growth in the first 5 h after inoculation, with growth rate ~1.3 h^−1^ corresponding to a doubling time of ~30 min in agreement with previous studies^58^ (Fig. 1b). Microscopy images of the liquid samples, taken in a capillary tube (see “Methods”), showed clearly the presence of multicellular aggregates coexisting with non-aggregated cells (Fig. 1c–e). At later times, when the culture ceased to grow exponentially, the aggregates disappeared, suggesting that they had dispersed (Fig. 1f).

### Aggregate size distribution changes with time as aggregates grow

**Fig. 2:**
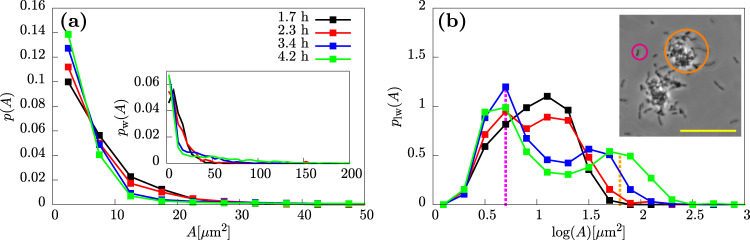
Aggregates increase in size during exponential growth. Distributions of aggregate sizes for PAO1 at four different times during incubation in LB. **a** The distribution of aggregate sizes. *p*(*A*) is the probability density of observing an aggregate of size *A*. Inset- weighted distribution, *p*_*w*_(*A*) in which the frequency is multiplied by the aggregate size. **b** Logged and weighted distributions, *p*_*lw*_(*A*), of aggregate sizes. *p*_*w*_(*A*) is the probability density for a pixel to belong to an aggregate of size *A*. Taking the log of the distribution has the effect of compressing the *x* axis which is necessary for visualisation given the broad range of aggregates in the system (see “Methods”). The inset shows a representative phase-contrast image of an aggregate and non-aggregated cells with orange and pink circles highlighting an aggregate and single cell respectively. Scale bar = 30 μm.

Visual inspection of microscopy images taken at different times (Fig. 1c, d) suggests that aggregates become larger over time during the exponential growth of a culture. To investigate this more quantitatively, we developed an automated image analysis protocol for measuring the cross-sectional area of individual aggregates on the bottom surface of the viewing capillary (see “Methods”), an approach similar to that of previous studies^57^. This allowed us to obtain a probability distribution of observed aggregate sizes, for each of our samples (Fig. 2a). The probability *p*(*A*) of observing an aggregate of cross-sectional area *A* is a decreasing function of *A*, indicating that small aggregates are more abundant than larger ones. Non-aggregated cells were the most abundant entities in all of our samples.

To highlight more clearly the changes in the aggregate size distribution with time, we computed the weighted distribution *p*_*w*_(*A*) (Fig. 2a, inset). This highlights the contribution of larger aggregates. It consists of the raw aggregate size probability distribution *p*(*A*) (Fig. 2a), multiplied by the aggregate size. Hence, *p*_*w*_(*A*) provides an estimate of the probability that a given bacterium in our sample belongs to an aggregate of size *A* (see “Methods”). To better visualise the contribution of the larger aggregates to the distribution, we also take the logarithm of the aggregate size before weighting to give the log-weighted distribution *p*_*lw*_(*A*) (Fig. 2b).

This form of analysis (Fig. 2b) clearly shows that, over time, a coexistence emerges between non-aggregated cells, to which we attribute the peak at $\log (A) \sim 0.7$ (*A* = 10^0.7^ = 5 μm^2^), and aggregates, to which we attribute the peak on the right side of the distribution $\log (A)\, >\, 1.5$ (*A* > 10^1.5^ = 30 μm^2^). The “aggregate peak” shifts to the right at later times (blue and green curve), indicating growth of the aggregates. In contrast, the non-aggregated-cell peak does not shift, consistent with our expectation that non-aggregated cells should not change in size during the exponential phase of growth.

### Both collisions and clonal growth are involved

**Fig. 3:**
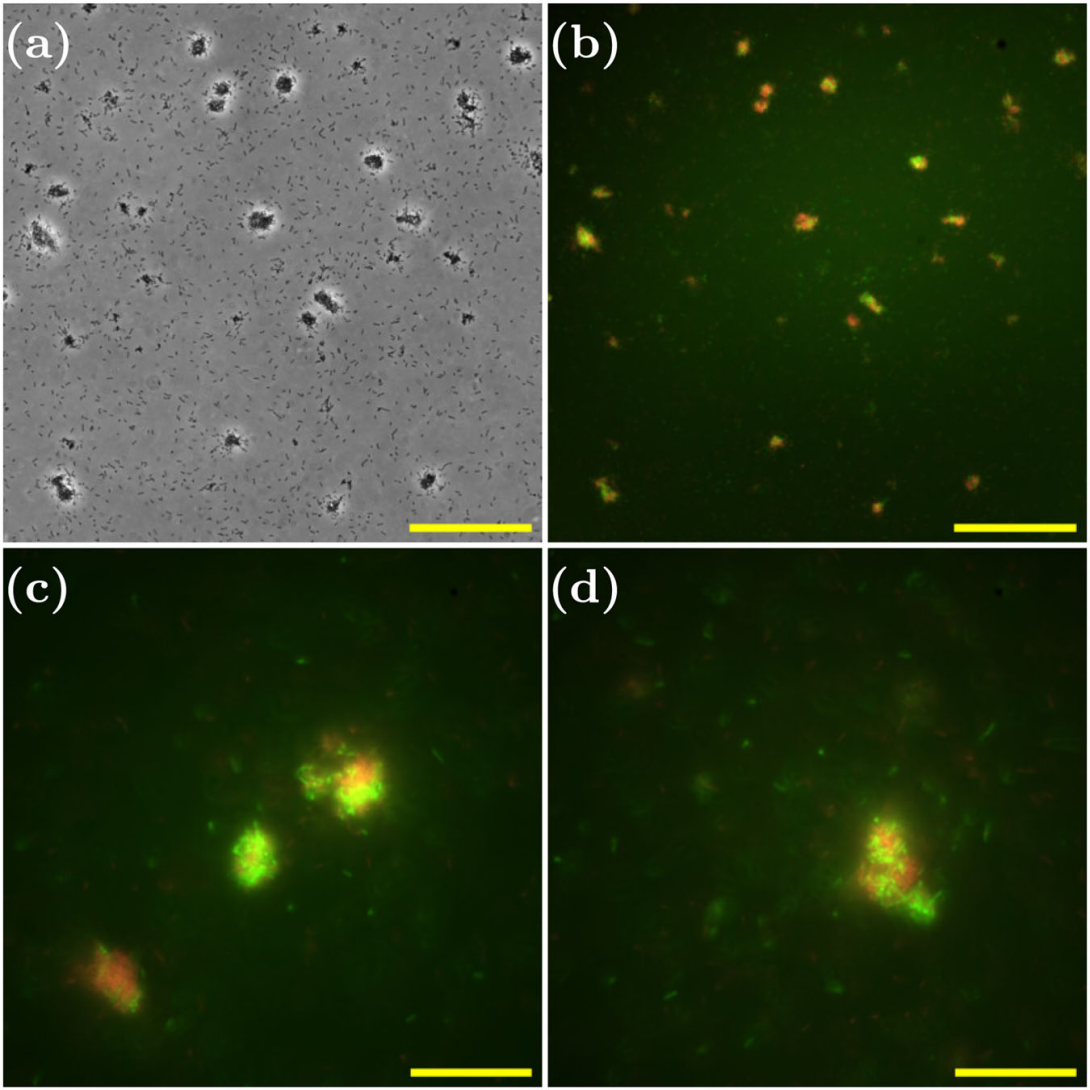
Cell–cell collisions are involved in aggregate growth. Phase-contrast and fluorescence microscopy images of aggregated samples of a mixed system of PAO1 GFP and PAO1 mCherry. **a** ×30 phase-contrast image at 4.8 h, scale bar = 100 μm. **b** Superposed fluorescence images from green (PAO1 GFP) and red channels (PAO1 mCherry) corresponding to (**a**), scale bar = 100 μm. **c**, **d** ×100 superposed fluorescence images from green (PAO1 GFP) and red channels (PAO1 mCherry) at 5.0 h, scale bar = 30 μm.

Growth of an aggregate could happen due to the proliferation of cells within the aggregate (clonal growth), or due to collisions and subsequent sticking of single cells or other aggregates from within the culture. Previous evidence for *P. aeruginosa*^7^ and for *Staphylococcus aureus*^52^ have suggested that both mechanisms might be involved. To assess the relative importance of these two mechanisms in the growth of our exponential phase aggregates, we repeated our experiment, this time inoculating with a 1:1 ratio (see “Methods”) of mCherry-labelled (red) and GFP-labelled (green) PAO1 cells (both from overnight cultures). If aggregation is mainly due to clonal growth, we would expect aggregates to be composed of cells of a single colour (reflecting the colour of the progenitor cell in each aggregate), while if growth by collisions predominates, we would expect to see a mix of colours within each aggregate. Using epifluorescence microscopy to image the aggregates that formed, we observed both red and green bacteria within the same aggregate (Fig. 3), suggesting that collision processes are indeed involved in aggregate growth. However, upon closer inspection, we found that the colour distribution within the aggregate was not entirely well mixed; instead, it consisted of red domains and green domains. This suggests that clonal growth is also occurring in the suspended exponential phase aggregates.

### Psl is responsible for exponential phase aggregation

**Fig. 4:**
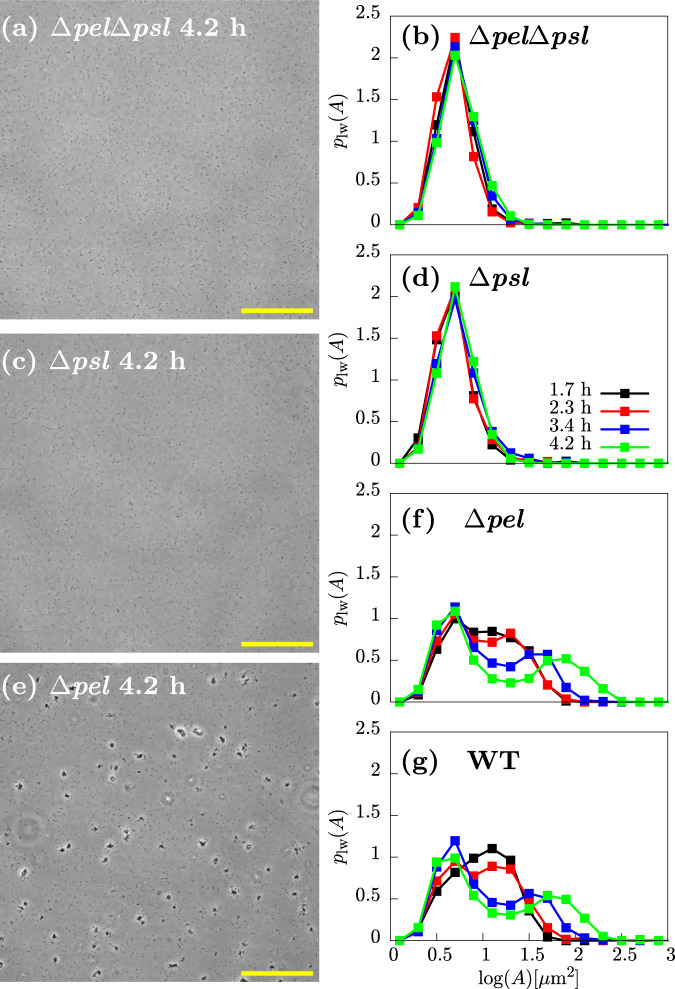
The Psl polysaccharide is required for aggregation. Representative images and distributions of aggregate sizes for the Δ*pel*Δ*psl*, Δ*psl*, Δ*pel* strains. **a**, **c**, **e** ×20 representative snapshots of the polymer mutants at 4.2 h. **b**, **d**, **f** Logged and weighted aggregate size distributions at various times. For comparison, the distribution for the WT is also shown (**g**). Scale bar = 150 μm.

To assess the involvement of the polysaccharides Pel and Psl in aggregate formation, we used three *P. aeruginosa* strains with different deficiencies in polysaccharide production: the mutant Δ*pel*, which can produce Psl but not Pel; the mutant Δ*psl*, which can produce Pel but not Psl, and the double mutant Δ*pel*Δ*psl*, which cannot produce either Psl or Pel. We observed no aggregates in samples taken from exponential cultures of the two non-Psl-producing strains Δ*psl* and Δ*pel*Δ*psl*; this was also reflected in the lack of an aggregate signal in the weighted size distribution *p*_*lw*_(*A*) (Fig. 4a–d). Therefore, these data suggest that Psl is essential for aggregate formation in our experiments.

The Δ*pel* strain, however, did aggregate (Fig. 4e). Furthermore, quantification of the log-weighted aggregate size distribution *p*_*lw*_(*A*) (Fig. 4f) produced results that were essentially identical to the wild-type (Fig. 4g). This suggests that Pel does not play a role in aggregate formation under these conditions.

**Fig. 5:**
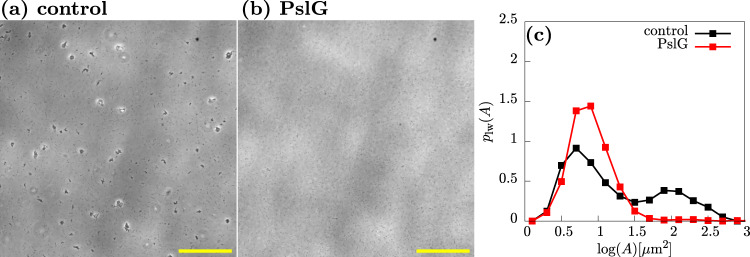
Psl is holding multicellular aggregates together. Representative phase-contrast microscopy images (×20) of a control sample (**a**) and a sample treated with the glycoside hydrolase PslG (**b**). **c** Aggregate size distributions for the control and PslG-treated samples. Samples were incubated for 3.7 h, PslG was added (see “Methods”), and samples were imaged 45 min later. Note there was a 20 min lag between imaging the control (4.1 h) and the enzyme-treated (4.4 h) samples. Scale bar = 150 μm.

To confirm that Psl is responsible for aggregate cohesion, we added the enzyme PslG, which degrades Psl^39,40^, to aggregated samples of the wild-type PAO1 strain. Addition of PslG (see “Methods”) results in loss of aggregates, as assessed both by microscopy (Fig. 5a, b) and by quantification of the log-weighted aggregate size distribution (Fig. 5c), in which the aggregate peak almost completely disappears in the presence of PslG. Since aggregates disappear when Psl is removed by the action of PslG, it appears that Psl provides a physical sticking force that holds the aggregates together.

### eDNA plays no role in exponential phase aggregation

**Fig. 6:**
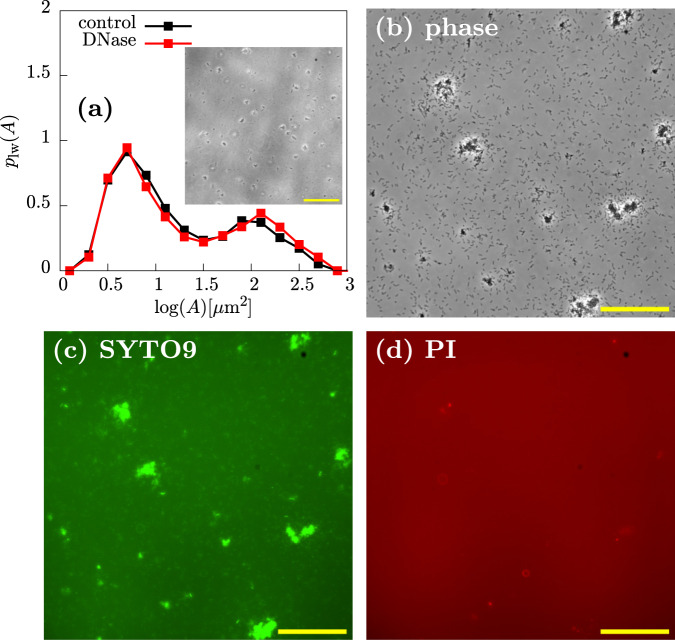
eDNA is not mediating aggregation. **a** Aggregate size distributions for control and samples treated with DNase at 3.7 h after inoculation (see “Methods”). Inset shows a representative phase-contrast image (4.1 h, ×20, scale bar = 150 μm) from which the red distribution (DNase treated) was computed. **b**–**d** Live-Dead staining of PAO1 cultures with SYTO9 (green) and PI (red) at 3.8 h after inoculation with dyes added 10 min before imaging (different data set to (**a**)). **b** ×30 phase-contrast image of representative snapshot shows aggregates and non-aggregated cells. **c** Corresponding image showing that many cells are stained with SYTO9 (green). **d** Corresponding image showing that few cells are stained with PI (red). Scale bar = 100 μm.

Previous work suggests that, in addition to Psl, eDNA can play a role in the aggregation of *P. aeruginosa* PAO1 (albeit in later-stage cultures)^6,7,16,17^. To test the role of eDNA in our exponential phase aggregates, we added DNase I, which degrades DNA, to aggregated samples from exponential cultures. DNase I had no effect on the aggregates in our samples, as observed by microscopy (Fig. 6a, inset). Furthermore, quantification of the log-weighted aggregate size distribution revealed no significant effect of DNase I treatment (Fig. 6a). This suggests that eDNA plays no role in the formation of exponential phase aggregates in our experiments.

We also stained aggregated samples from exponential phase cultures with the dyes SYTO-9 (green) and propidium iodide (PI, red) (Fig. 6c, d). SYTO-9 traverses the cell membrane and binds chromosomal DNA, so it can be used to locate cells in a fluorescence image. PI is a DNA-binding dye that does not traverse intact bacterial membranes, so it can be used to visualise eDNA, although dead cells will also appear as intense objects^16^. We observed little signal in the red (PI) channel (Fig. 6b–d; contrast to stationary phase aggregates in Fig. 9), suggesting that exponential phase aggregates contain few dead cells, and little eDNA.

### Aggregation is mediated by cell-associated Psl

Our results imply that Psl mediates aggregation in exponential phase cultures of PAO1, with little or no role for either eDNA or Pel. However, Psl exists in two forms: a cell-free form that is secreted into the medium, and a cell-associated form that remains bound to the cell surface^36,37^.

**Fig. 7:**
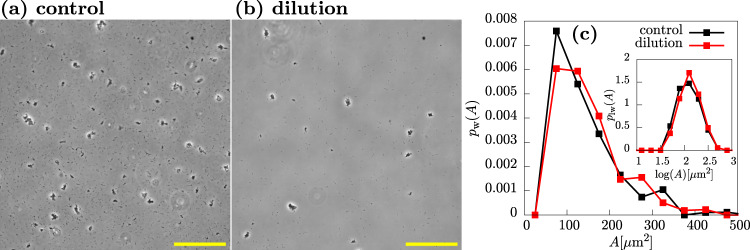
Aggregate size is unaffected by dilution. Upon dilution, the number of cells and aggregates in the field of view is decreased but aggregate size is unaffected. **a** Representative phase-contrast microscopy image (×20) of control sample of PAO1 at 3.6 h after inoculation. **b** Phase-contrast image (×20) of diluted sample at 3.6 h after inoculation. (Note sample was incubated for 3.6 h. Then the sample was diluted and imaged 5 min later). **c** Weighted distributions and log-weighted distributions (inset). Here, we are concerned with how dilution affects the aggregate size, therefore entities less than 50 μm are not considered in the construction of the distributions. Scale bar = 150 μm.

To gain insight into the role of cell-free vs. cell-associated Psl, we reasoned that if cell-free Psl, released into the medium, is important for cohesion, then diluting the culture should compromise the aggregates, since it will reduce the concentration of cell-free Psl that is present. In contrast, if aggregates are held together by cell-associated Psl, they should remain intact after dilution of the culture, since the Psl is bound to the surfaces of the cells within the aggregate. Upon diluting aggregated samples from an exponential culture with fresh medium, we observed, as expected, a decrease in the observed numbers both of aggregates and single cells (Fig. 7a, b). However, quantification of the weighted aggregate size distribution (Fig. 7c) revealed no difference in the size of the aggregates (Fig. 7a, b). This suggests that aggregate cohesion is mediated by cell-associated rather than cell-free Psl.

### Aggregation is specific to Psl-producing cells

To further test the hypothesis that cell-associated Psl mediates cohesion, and to understand its socio-evolutionary implications, we investigated a mixed system of green Psl producers (Δ*pel*-GFP) and red non-producers (Δ*pel*Δ*psl*-mCherry). In particular, we asked whether Psl non-producers could join aggregates of Psl producers.

**Fig. 8:**
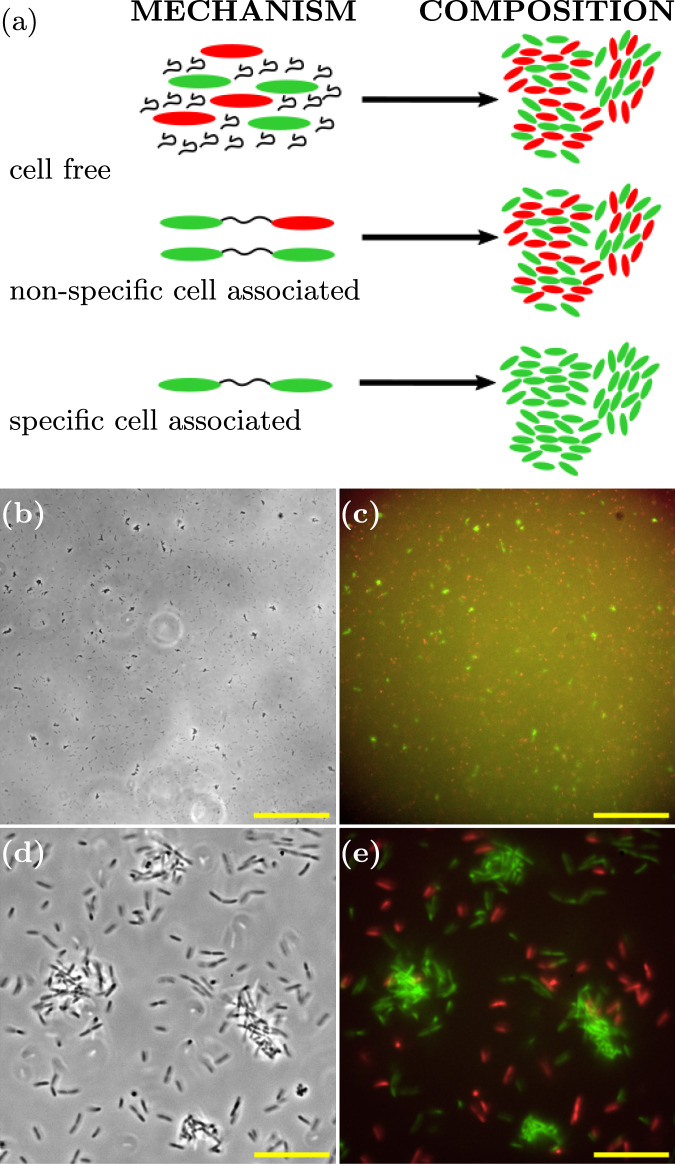
Cell-associated Psl mediates cell–cell cohesion. **a** Schematic outlining the composition of aggregates (right) resulting from the corresponding mechanism of Psl-mediated cohesion (left). **b** Representative phase-contrast microscopy image (×20 magnification) taken 3.8 h after inoculation, scale bar = 150 μm. **c** Superposed fluorescence images from green (Psl-producer, Δ*pel*-GFP) and red (non-producer, Δ*pel*Δ*psl*-mCherry) channels corresponding to (**b**), scale bar = 150 μm. **d** Representative phase-contrast microscopy image (×150 magnification) 4.0 h after inoculation, scale bar = 20 μm. **e** Superposed fluorescence images from green (Psl-producer, Δ*pel*-GFP) and red (non-producer, Δ*pel*Δ*psl*-mCherry) channels corresponding to (**d**), scale bar = 20 μm.

If aggregation were mediated by cell-free Psl, we would expect the Psl to be shared between producers and non-producers, since it is secreted into the medium. Therefore one would expect to see mixed red-green aggregates, composed of both producers and non-producers (Fig. 8a).

If, on the other hand, the cell-associated form of Psl mediates aggregation, then we might expect either mixed aggregates or producer-only aggregates. Mixed aggregates are expected if cell-associated Psl binds non-specifically, such that it can form a “bridge” between a producer and a non-producer. Producer-only aggregates are expected if cell-associated Psl is specific in its binding, forming bridges only between producer cells (Fig. 8a).

Fluorescence microscopy of the mixed culture revealed that aggregates consisted only of Psl-producing (green) cells (Fig. 8b–e). This confirms that, indeed, cohesion is mediated by cell-associated Psl, and furthermore, the interaction is specific to Psl producers: Psl associated with a producer cell leads to a cohesive interaction only with other producer cells, not with non-producer cells.

Previous research has shown that Psl can bind specifically to the matrix protein CdrA^32,59^; we therefore speculated that the cell-associated Psl interaction that we observe might involve CdrA. However, we observed no discernible difference in aggregation for a mutant deficient in CdrA (Supplementary Fig. 1).

### In stationary phase cultures, both eDNA and Psl mediate aggregation

**Fig. 9:**
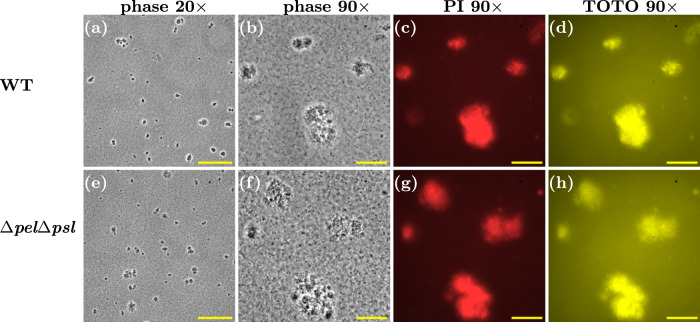
Aggregates of WT and Δ*pel*Δ*psl* strains are present in stationary phase culture. **a**–**d** Representative snapshots of WT aggregates in overnight culture. **a** Phase contrast ×20 magnification. **b** Phase contrast ×90 magnification. **c** Red fluorescence channel, corresponding to image in (**b**), highlighting PI staining to extracellular DNA or intracellular DNA if cell membrane is compromised. **d** Yellow fluorescence channel, corresponding to image in (**b**), highlighting TOTO staining to extracellular DNA or intracellular DNA if cell membrane is compromised. **e**–**h** Δ*pel*Δ*psl* snapshots corresponding to (**a**–**d**). Scale bar = 150 μm in (**a**) and (**e**). Scale bar = 30 μm in (**b**–**d**) and (**f**–**h**).

Previous work has indicated a role for eDNA, or eDNA and Psl, in the aggregation of *P. aeruginosa* PAO1 in late-log or stationary phase cultures^6,7,16,17^. We also obtained microscopy images of samples from overnight, stationary phase cultures. Aggregates were clearly visible in these samples (Fig. 9a, b), and these aggregates were stained by both PI and TOTO, indicating the presence of eDNA and/or dead cells (Fig. 9c, d) (TOTO, like PI, is a DNA-binding dye that does not traverse intact bacterial membranes, so can be used to visualise eDNA as well as dead cells^16^).

**Fig. 10:**
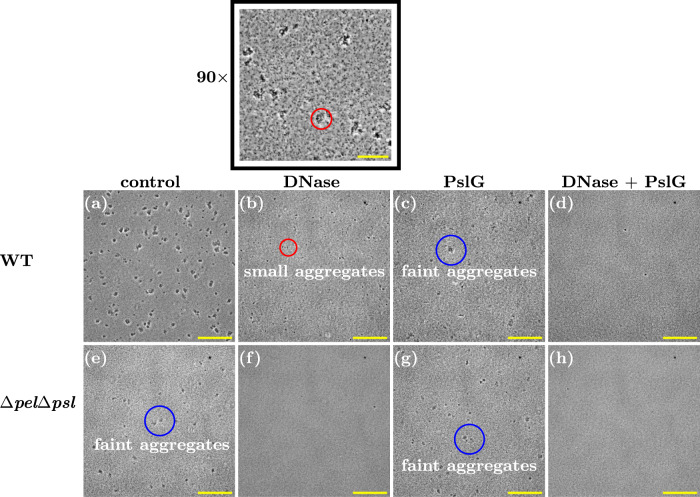
Both Psl and eDNA mediate aggregation in overnight stationary phase cultures. **a**–**d** WT strain subjected to treatment with DNase and PslG. **e**–**h** Δ*pel*Δ*psl* strain subjected to treatment with DNase and PslG (×20 magnification). Small and faint aggregates are highlighted with red and blue circles, respectively. Scale bar = 150 μm. The black-boxed image corresponds to the same sample as (**b**) with ×90 magnification, scale bar = 30 μm.

To confirm the role of eDNA and/or Psl in these stationary phase aggregates, we treated aggregated samples with DNase I (to degrade eDNA) or PslG (to degrade Psl), or both. Treatment with either DNase I or PslG resulted in smaller and less easily visible aggregates (compare Fig. 10a–c). Simultaneous treatment with both enzymes led to the complete loss of the aggregates. Therefore both eDNA and Psl are implicated in stationary phase aggregate formation in the PAO1 strain.

Interestingly, the double polymer knockout strain Δ*pel*Δ*psl*, which cannot produce either Psl or Pel, also forms aggregates in overnight stationary phase culture, although these have a somewhat different appearance to those of the wild-type, appearing fainter in phase-contrast images (Figs. 9e, f and 10e), similar to the wild-type aggregates after treatment with PslG (compare Fig. 10e to Fig. 10c). The stationary phase Δ*pel*Δ*psl* aggregates stain with PI and TOTO (Fig. 9g, h), suggesting that their cohesion is provided by eDNA. Indeed, treating these aggregates with DNase I led to their complete disappearance (Fig. 10f, h), while treating them with PslG had no effect (Fig. 10g).

### Stationary phase aggregates do not seed exponential phase aggregates

Kragh et al. hypothesised a “snowball” mechanism, in which pre-existing small aggregates, present in the culture inoculum, could act as seeds for aggregate growth^7^. Since our exponential phase cultures are inoculated from overnight stationary phase inocula that contain DNA and Psl-mediated aggregates (Fig. 9), we wondered whether these pre-existing aggregates might act as seeds for the Psl-mediated aggregates that we observe in the exponential phase. In other words, exponential phase aggregates might not form de novo but rather via Psl-mediated growth of pre-existing small aggregates that were already present in the inoculum.

To test this, we inoculated exponential cultures with stationary phase inocula that had been pre-treated by mechanical dispersion using a syringe, breaking up all aggregates (see “Methods” and Supplementary Fig. 2a–c). This system is thus “unseeded”, since there are no aggregates present in the inoculum. This aggregate-removal pre-treatment had no effect on our results (Supplementary Fig. 2d, e), suggesting that the aggregates which we observe in the exponential phase are not seeded from pre-existing aggregates in the stationary phase inoculum but rather form de novo during exponential growth.

## Discussion

In this work, we investigated quantitatively the formation of suspended aggregates in exponentially growing liquid cultures of *P. aeruginosa* PAO1, compared to overnight stationary-phase cultures. We observe distinct types of aggregates in different growth stages of the cultures. In exponential cultures, aggregation is mediated by Psl alone, aggregates appear de novo, and they grow via a mixture of collisions and clonal growth. Psl-mediated aggregation is specific to Psl-producers, since Psl non-producers do not aggregate with producers. In contrast, in overnight stationary phase cultures both Psl and eDNA are involved in aggregation, although eDNA alone is sufficient.

Our results are consistent with previous observations that both eDNA and Psl are important for aggregation in overnight stationary phase aggregates^6,7,16,17^. Our finding that Psl mediates aggregation in exponential phase cultures is also consistent with previous work^7^, however here we also show that it is cell-associated rather than cell-free Psl that plays the key role. Although previous studies have differentiated the roles of cell-associated and cell-free Psl in biofilm formation^25,38–40^, in our study, we differentiate between the two forms in planktonic aggregates.

The extent to which the physiological state of bacteria in liquid-phase aggregates mirrors the surface-attached biofilm state has been a topic of discussion^6,7,17^. In biofilms of *P. aeruginosa* PAO1, Psl has been found to play a role in initial attachment to surfaces^34,35,60,61^, microcolony and macrocolony formation^60^, and is thought to be crucial in forming the matrix that holds cells together in more mature biofilms. eDNA, on the other hand, is mainly associated with late-stage biofilm development^29^, although it has also been found to be relevant for structural stability^34^ and biofilm establishment^30,31^. For liquid-phase aggregates, our findings (and those of others^6,7,16,17^) that Psl and eDNA play a role in the later stages, and only Psl at earlier times, suggest that there might be parallels between the development of biofilms and suspended aggregates.

In this work, we observed no role for Pel in aggregation of PAO1 in liquid. Although we focused on the PAO1 strain in the present study, this finding might very well be different for other *P. aeruginosa* strains. For example, Pel has been implicated in biofilm formation in *P. aeruginosa* PA14^37,41^, as well as in aggregate formation in cystic fibrosis sputum^62^. Therefore, it would be interesting to investigate whether Pel is important for planktonic aggregation for strains other than PAO1, including clinical isolates. Previous work using atomic force microscopy suggests that Pel mediates cell–cell cohesion over a shorter range than Psl^35^. Therefore we might expect Pel-mediated aggregation to proceed rather differently to the Psl-mediated aggregation studied here.

Interestingly, and in common with other authors^7,17^, we observe a population of non-aggregated cells that coexist with multicellular aggregates, even at late times (Fig. 1d, e). This seems somewhat surprising, since naively, we might expect that non-aggregated cells would eventually attach to aggregates. In future work, it would be interesting to investigate the origin of this coexistence. One explanation might be that the non-aggregated cells are phenotypically different from those in aggregates, for example, they produce less Psl. This in turn might be connected to the positive feedback regulatory circuit found in *P. aeruginosa* biofilms where Psl production stimulates increasing c-di-GMP which in turn stimulates increased Psl production^63^, potentially leading to bistability in Psl production. Alternatively, the non-aggregated cells in our cultures might originate from dispersal of previously formed aggregates. Future experiments aiming to track the fate of individual cells during aggregate formation and dispersal, while challenging, would be very valuable. Other, more general questions, might also arise, such as how aggregation proceeds in suspensions in which cell–cell cohesion (“stickiness”) depends on the local cell density^64^.

Previous work has established that, in biofilms, Psl production is a social trait, in that the benefits of Psl are shared between cells, but it is relatively non-exploitable, in that Psl non-producers do not outcompete Psl producers within a biofilm^43^. In the biofilm context, Psl non-producers obtained social benefits from Psl producers, since they were better able to attach to a surface and form biofilm in the presence of producers^43^. In our study, however, Psl non-producers do not co-aggregate with Psl producers, suggesting that non-producers are unlikely to gain any benefit from producers. Our results therefore hint that the social role of Psl might be different in suspended aggregates compared to surface-attached biofilms. In future work, it would be interesting to measure systematically the fitness benefits of aggregation for producers and non-producers in mixed cultures.

An innovative aspect of the work presented here is that we quantify the distribution of aggregate sizes. This allows us to determine, for example, that aggregates are indistinguishable for Psl-producers and non-producers. Quantification of the aggregate size distribution is also an important step towards the development of mathematical and computational models for bacterial aggregation^64–71^, similar to those that have been established for many years in the field of colloid science^72–79^. In particular, it is well established in the field of colloidal-polymer suspensions that micron-sized colloidal particles can aggregate either by depletion interactions, in which free-floating polymers in the suspension exert osmotic forces that push the colloids together^80,81^, or by polymer bridging interactions, where polymer molecules directly bind to the surfaces of the colloidal particles, sticking them together^82,83^. Depletion interactions have been implicated in several cases for bacterial aggregation in liquid suspension^84–86^ and for host-polymer bacterial interactions in chronic infections^87–89^ as well as experimental^90^ and simulated^91^ biofilms. In contrast, here we explicitly show that depletion is not relevant in exponential phase aggregation of *P. aeruginosa* PAO1, since the aggregates remain unchanged when we dilute the culture. Instead, we show that cell-associated Psl is responsible, suggesting a direct bridging interaction. Aside from the recent work of Jenning et al.^62^ which suggested the involvement of a polymer bridging interaction in CF sputum aggregates, to our knowledge, our study is the first to point to a polymer bridging interaction in the context of planktonic aggregates. It will be important in the future to understand the molecular mechanisms behind this bridging, and subsequently construct appropriate models that can predict, for example, the time taken for a culture to aggregate.

Although quantitative measurements of aggregate size distributions using microscopy are valuable, they are challenging in several ways. Our distribution *p*(*A*) is in fact the probability of observing an aggregate of area *A* on the bottom surface of a capillary (containing the liquid suspension) when viewed from beneath with an inverted microscope. Similar to previous studies^57^, we use the area of the aggregates projected onto the surface as a proxy for aggregate size. Although this is not a true aggregate size distribution, owing to the fact the aggregates are three-dimensional entities, it nevertheless allows one to compare aggregates at different stages during growth and also to compare aggregates between different strains. Combining this type of analysis with high-throughput methods, like those used to assess coaggregation in aggregates of oral bacteria^92^, will allow for a better understanding of aggregation in *P. aeruginosa* and in other strains that are clinically and industrially relevant.

Suspended bacterial aggregates are so ubiquitous that they have been proposed to constitute a third mode of growth for bacteria, along with the biofilm and planktonic lifestyles^93^. Yet, the findings presented here highlight the fact that the physical, and perhaps also physiological properties of bacteria in suspended aggregates might be very different depending on the environment in which the aggregates form. Here, we conducted aggregation experiments for one strain (PAO1) in one chosen nutrient media (LB), yet nevertheless observed considerable complexity, with distinct aggregation behaviours for an exponentially growing culture compared to a stationary phase culture. Previous studies on aggregation of PAO1 have used a variety of media (LB^7^, glucose^6,9,16,17^, succinate, and acetate^17^), and have observed differences in the mechanism of aggregation—suggesting that finding universality in the mechanisms of aggregation, even for a single strain, may be highly nontrivial. Finding universal principles that apply to aggregation across a diverse range of bacterial species in a diverse range of environments (e.g., marine snow vs lake snow) is an even more challenging task^93^. Nevertheless, progress towards a more complete picture can be achieved by investigating aggregation under different growth conditions and with multiple (and mixed) strains. Although such an endeavour is likely beyond the scope of any one single study, extending the research presented here can increase our understanding of how the physical and biological properties of planktonic aggregates depend on their formation pathways, and the possible consequences of this for seeding of surface-attached biofilms^9,10^, as well as for antibiotic susceptibility in clinical infections, control of aggregation pathways and, potentially, our understanding of the evolution of multicellularity more generally.

## Methods

### Growing liquid cultures

*Pseudomonas aeruginosa* strains (Table 1) were grown overnight from frozen stock cultures (80 °C, 80% overnight cultures in LB and 20% glycerol) in 5 mL of Miller Lysogeny Broth (LB) at 37 °C and 180 rpm for up to 16 h. Fresh culture was inoculated with a 1:1000 dilution of the overnight culture (100 μL of the overnight culture into 100 mL of fresh LB in a 500 mL Erlenmeyer flask). This is referred to as time zero (*t* = 0) for our exponential phase growth experiments. The freshly inoculated culture was incubated at 37 °C and shaken at 180 rpm in an orbital incubator (Stuart, UK). The growth of the culture was monitored by taking OD_600_ measurements using a Cary 100 UV–Vis spectrophotometer (Agilent Technologies, USA).

**Table 1:** Strains used in this study.

Strain	Description	Source
PAO1 Δ*pel*	Pel-knockout mutant	^59^
PAO1 Δ*psl*	Psl-knockout mutant	^95^
PAO1 Δ*pel*Δ*psl*	Pel- and Psl-knockout mutant	^59^
PAO1 mCherry	Red-fluorescent strain with chromosomally integrated constitutive mCherry gene	^96,97^
PAO1 GFP	Green-fluorescent strain with chromosomally integrated constitutive mCherry gene	^96,97^
PAO1 Δ*pel*-GFP	Pel-knockout mutant with chromosomally integrated constitutive GFP gene	^43^
PAO1 Δ*pel*Δ*psl*-mCherry	Pel- and Psl-knockout strain with chromosomally integrated constitutive mCherry gene	^43^

### Imaging aggregates formed in liquid cultures

Samples were loaded into borosilicate glass capillaries (Vitrocom, 0.4 × 8.0 × 50 mm, volume ~180 μL). The chamber was sealed using petroleum jelly to prevent leakage, and then placed onto a fully automated inverted microscope (Nikon TE300 Eclipse, Hamamatsu ORCA-Flash 4.0 camera) for imaging using phase-contrast and fluorescence microscopy. Once placed under the microscope, snapshots were taken at multiple horizontal (xy) positions on the capillary floor. Three types of objectives were used: a Nikon 20×/0.5 objective; a Nikon extra-long-working distance 60×/0.7 objective; and a Nikon 100×/1.3 oil objective. For the higher resolution images (×100 magnification) a different procedure was used. Instead of using a capillary tube, a small enclosure was created by attaching a gene frame to a microscopy slide. In total, 100 mL of samples were loaded into this enclosure, which was then sealed with a cover slip, inverted, and placed onto the microscope for imaging.

Imaging at magnifications ×30, ×90, and ×150 was achieved using the microscope’s in-built 1.5 × magnifier on the ×20, ×60, and ×100 objectives, respectively. Pixel dimensions of 2048 × 2048 (1 × 1 binning) were used for imaging in phase contrast alone, whilst dimensions of 1024 × 1024 (2 × 2 binning) were used for combined fluorescence and phase-contrast imaging.

### Syringing cultures

To assess the effect of aggregates in the initial inoculum, 1 mL sample of overnight culture was vigorously passed through a 20 G needle (BD, Spain) five times with a 1-mL syringe (BD, Spain). This was the control sample.

### Preparation and imaging of suspensions of PAO1 GFP and PAO1 mCherry strains

Suspensions were prepared by adding 25 μL of an overnight culture of PAO1 GFP (OD = 3.0) and 75 μL of an overnight culture of PAO1 mCherry (OD = 1.0) to 100 mL of fresh LB. Cultures were grown and imaged according to the methods outlined above. The dsRED channel (excitation FF01-554/23, emission FF01-609/54), and GFP channel (excitation FF01-474/27, emission FF01-525/45), were used for visualisation of the mCherry and GFP strains, respectively.

### Computing the distribution of aggregate sizes

Phase-contrast microscopy images (Supplementary Fig. 3, left) were processed using FIJI^94^ to generate the corresponding binary images (Supplementary Fig. 3, right). First, a rolling ball background subtraction (FIJI, in-built function) was applied to correct for an unevenly illuminated background. Here we used a rolling ball radius of 10 pixels. Binary images of dark object on light backgrounds were then generated with FIJI’s in-built threshold function using default and automatic settings. The resulting area of each dark object in each frame was then computed using FIJI’s analyse particle functions. This process was performed on many (~10–40) microscopy images representing different fields of view (xy positions) in the sample.

In order to compute an area distribution, the outputted area sizes were used as input data for our in-house software (written in Fortran 90). Although it is somewhat arbitrary, we only considered aggregates with an area greater than 2 μm^2^. This is because visual inspection reveals that cells on the surface tend to have an area greater than 2 μm^2^ (Supplementary Fig. 3; see also the individual cell peak in Fig. 2 at $\log (A) \sim 0.7$ (10^0.7^ = 5 μm^2^)). The software then builds the distribution, *p*(*A*) by counting the number of aggregates *n*_*A*_ of area *A*

1 $$p(A)=\frac{{n}_{A}}{N}.$$

*N* is the total number of aggregates in the system and provides the normalisation

2 $$\mathop{\sum}\limits_{A}p(A)=1,$$

where the sum is over all aggregate sizes. We also computed a weighted distribution *p*_*w*_(*A*) given by

3 $${p}_{w}(A)=\frac{{n}_{A}A}{{A}_{T}},$$

where *A*_*T*_ is the total area of aggregates in the system. Here we see that the number of aggregates *n*_*A*_ of size *A* is multiplied by *A* (weighted) to ensure that larger aggregates make a greater contribution to the distribution. The presence of *A*_*T*_ in the denominator ensures *p*_*w*_(*A*) is normalised according to

4 $$\mathop{\sum}\limits_{A}{p}_{w}(A)=1.$$

Given the large range of aggregate sizes observed in the system (2–1000 μm^2^), we also generated distributions in which the logarithm of the area (${\log }_{10}(A)$) was first computed before the frequency of the area is weighted by the area size. This has the effect of compressing the *x* axis, thus making for easier visualisation.

Note that the black spot in the upper right corner of the microscopy images is a camera artefact and is subtracted from the binary images before the computation of the aggregate size distributions.

### Enzyme treatment of exponentially growing cultures

For PslG treatment, 22 μL of 4.5 mg/mL of PslG solution^38^ was added to a 20 mL universal flask along with 1 mL of liquid culture, which had been incubated for 3.7 h. The culture was then incubated for a further 40 min at 37 °C while shaken at 180 rpm. For DNAse treatment, 100 μL of 100 μg/mL of DNase 1 (STEMCELL technologies, Canada) was added to 0.9 mL of liquid culture (incubated for 3.7 h) in a 20 mL universal flask. The culture was then incubated for a further 30 min at 37 °C while shaken at 180 rpm. Samples were then visualised under the microscope. As a control, 1 mL of liquid culture (incubated for 3.7 h) was added to a 20-mL universal flask, without enzyme, and incubated for a further 30 min at 37 °C while shaken at 180 rpm.

### Staining and fluorescence imaging of exponentially growing cultures

For propidium iodide (PI)/Syto9 imaging, 20 μL of 100 μg/mL PI (Thermo Fisher) and 20 μL of 100 μM Syto9 (Thermo Fisher) were added to a 20-mL universal flask along with 2 mL of exponential phase culture, which had been incubated for 3.5 h. The sample was left for 15 min before being loaded into a capillary and imaged under the microscope. The dsRED channel (excitation FF01-554/23, emission FF01-609/54), and GFP channel (excitation FF01-474/27, emission FF01-525/45) were used for visualisation of the PI and SYTO9 dyes, respectively.

### Dilution of exponential phase cultures

Exponential phase cultures were visualised under the microscope to check for the presence of aggregates in the bacterial suspension. After 3.5 h of growth, 120 μL of the exponential phase culture was added to 1080 μL of fresh LB media in a universal flask to give a dilution factor of 1:10. As a non-diluted control, 1200 μL of the exponential phase culture was added to a universal flask. Both samples were left to sit for 10 min before being visualised under the microscope.

### Preparation and imaging of suspensions of Δ*pel* GFP and Δ*pel*Δ*psl* mCherry

Suspensions were prepared by adding 50 μL of an overnight culture of Δ*pel* GFP (OD = 1.9) and 50 μL of an overnight culture of Δ*pel*Δ*psl* (OD = 1.5) to 100 mL of fresh LB media to give a total dilution of 1:1000. The mixed culture was then incubated and images acquired as described above.

### Staining and fluorescence imaging of stationary phase cultures

For staining, 10 μL of 100 μg/mL Propidium Iodide (PI) (Thermo Fisher) plus 50 μL of 100 μM TOTO (Thermo Fisher) were added to 1 mL of stationary phase culture of WT cells (which had incubated for 22 h) in a 1 mL Eppendorf tube. The sample was left for 15 min before loading a capillary and imaged under the microscope. The dsRED channel (excitation FF01-554/23, emission FF01-609/54), and YFP channel (excitation FF01-500/24, emission FF01-542/27) were used for visualisation of the PI and TOTO dyes, respectively.

### Enzyme treatment of stationary phase cultures

In all, 30 μL of 4.5 mg/mL of PslG was added to 0.5 mL of stationary phase culture that had been incubated for 21 h. In total, 100 μL of 100 μg/mL of DNase 1 (STEMCELL Technologies, Canada) was added to 0.5 mL of stationary phase culture. As a control, 130 μl of PBS buffer was added to 0.5 mL of stationary phase culture. After the addition of enzymes, samples were left for 30 min at room temperature before being loaded into capillaries and imaged under the microscope.

### Reporting summary

Further information on research design is available in the Nature Research Reporting Summary linked to this article.

## Supplementary information

Supplementary material

Reporting Summary

## Data Availability

Experimental datasets generated are available from the corresponding author upon request.

## Code availability

Analysis code used during the current study are available from the corresponding author upon request.
