# Supplementary material for: Distinct types of multicellular aggregates in Pseudomonas aeruginosa liquid cultures
Source: NPJ Biofilms Microbiomes. 2023 Jul 28;9:52. doi: 10.1038/s41522-023-00412-5 (PMC10382557; doi:10.1038/s41522-023-00412-5)
Supplement: Supplementary file 1 — Supplementary material [file 41522_2023_412_MOESM1_ESM.pdf]

Supplementary Information - Distinct types of multicellular aggregates in *Pseudomonas aeruginosa* liquid cultures

Gavin Melaugh*, Vincent Martinez, Perrin Baker, Preston Hill, P. Lynne Howell, Daniel J. Wozniak and Rosalind J. Allen

*Corresponding author(s). E-mail(s): g.melaugh@ed.ac.uk;

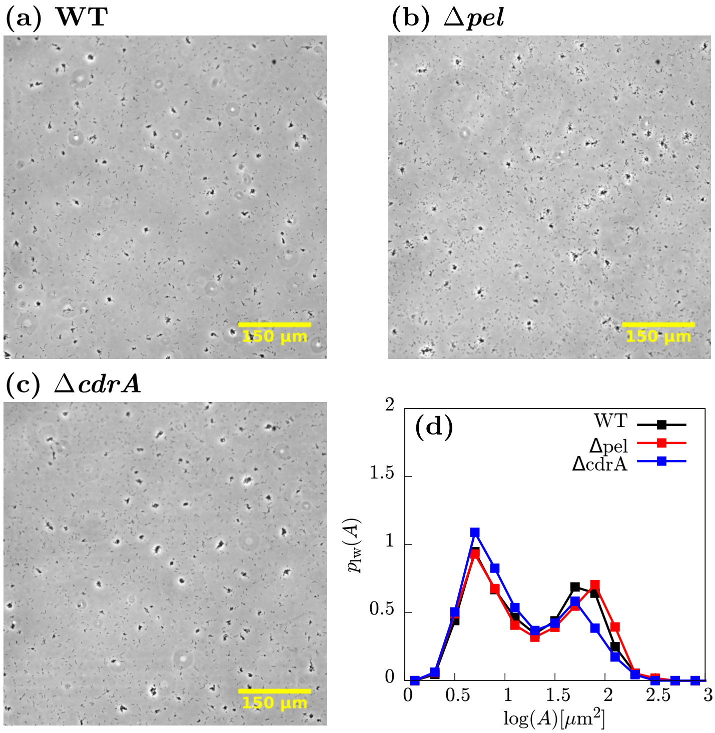

Supplementary Figure 1 - No obvious role of CdrA. Representative phase contrast microscopy images for (a) WT, (b) Δ*pel*, and (c) Δ*cdrA* at 2.5 h. (d) Corresponding aggregate size distributions.

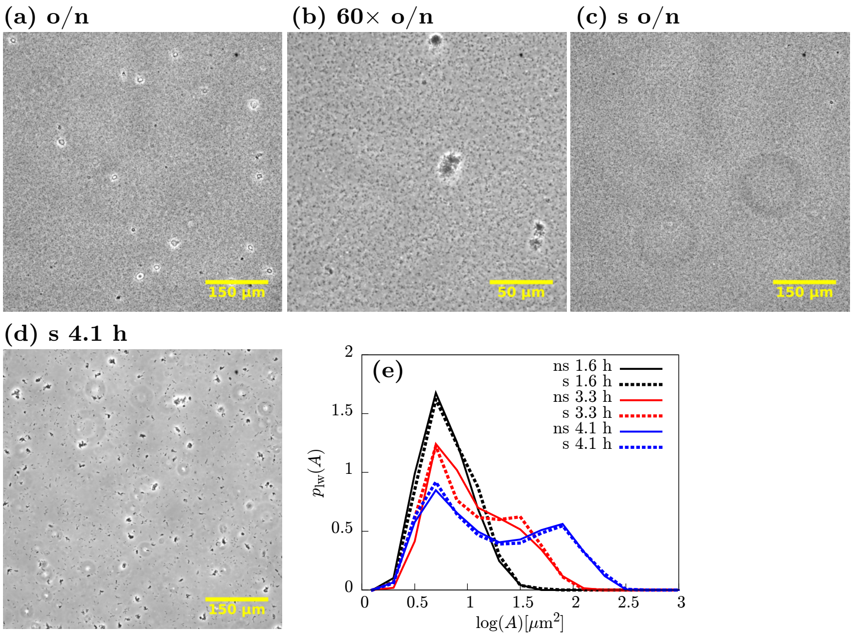

Supplementary Figure 2 - Exponential phase aggregates are not seeded by stationary-phase aggregates. (a) Phase contrast image showing aggregates in the overnight stationary phase culture (20×). (b) 60× magnification of overnight stationary phase culture in (a). (c) Phase contrast image of overnight stationary phase culture after mechanical disruption with syringe (20×). (d) Phase contrast image of exponentially growing culture that was innoculated with a mechanically disrupted overnight culture (20×). (e) Distribution of aggregate sizes in the exponential-phase cultures that were incoculated with (dashed lines) and without (solid lines) mechanically disrupted overnight (o/n) cultures. s and ns denote syringed and nonsyringed inocula respectively.

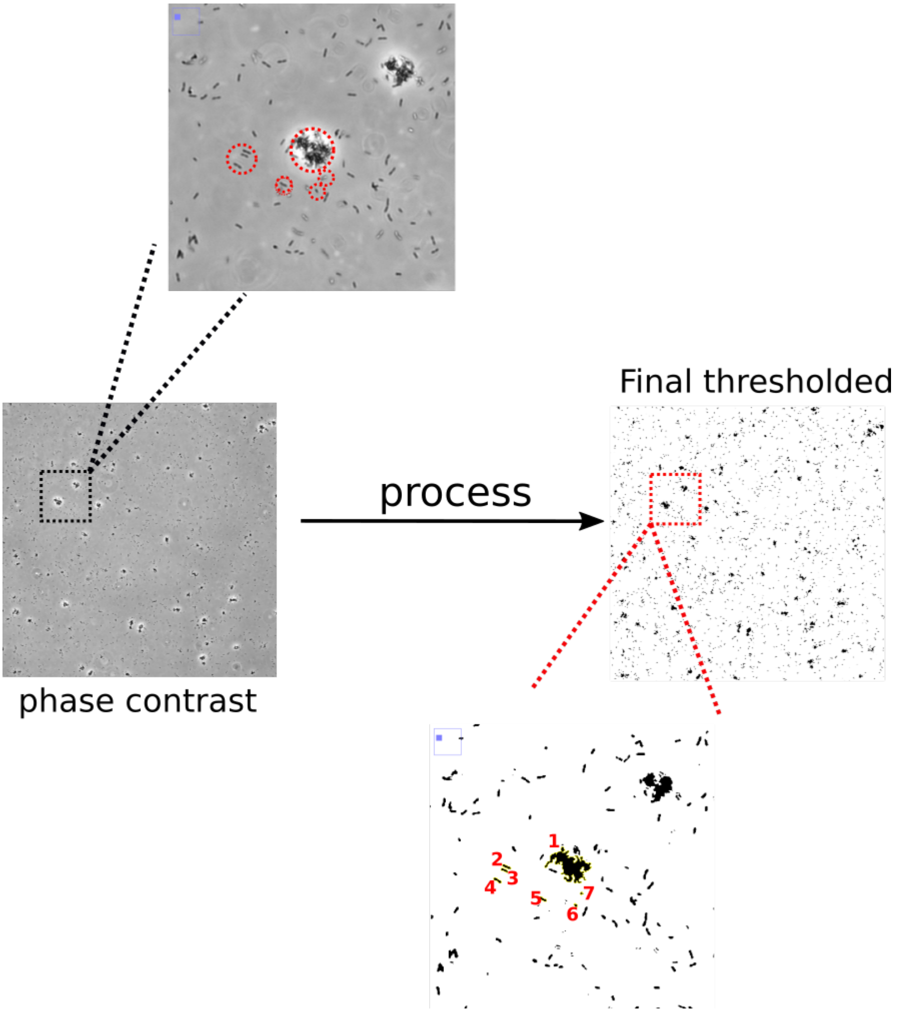

Supplementary Figure 3 - Computing the aggregate size distribution. Left: Representative phase contrast microscopy image of an aggregated sample. Right: Corresponding thresholded image after processing. In the zoomed-in-region of the phase contrast image, the red circles highlight particular groups of unaggregated cells as well as aggregates. The sizes of the cells/aggregate (entities) within these red circles, numbered from 1 to 7 are: 180, 5.28, 4.12, 5.39, 4.65, 2.23, and 1.48 μm^2 respectively
